# Partitioning the regional and local drivers of phylogenetic and functional diversity along temperate elevational gradients on an East Asian peninsula

**DOI:** 10.1038/s41598-018-21266-4

**Published:** 2018-02-12

**Authors:** Jung-Hwa Chun, Chang-Bae Lee

**Affiliations:** 1 Division of Forest Ecology, National Institute of Forest Science, 57 Hoegiro, Dongdaemungu, Seoul, 02455 Republic of Korea; 2 Global Resources Division, Korea Forestry Promotion Institute, 475 Gonghangdaero, Gangseogu, Seoul, 07570 Republic of Korea

## Abstract

Species-centric approaches to biodiversity in ecological research are limited in their ability to reflect the evolutionary history and functional diversity of community assembly. Recently, the introduction of alternative facets of biodiversity, such as phylogenetic and functional diversity, has shed light on this problem and improved our understanding of the processes underlying biodiversity patterns. Here, we investigated the phylogenetic and functional diversity patterns of α, β and γ components in woody plant assemblages along regional and local elevational gradients in South Korea. Although the patterns of phylogenetic and functional diversity varied along regional and local elevational transects, the main drivers were partitioned into two categories: regional area or climate for phylogenetic diversity, depending on whether the transect was at a regional or local scale; and habitat heterogeneity for functional diversity, which was derived in elevational bands. Moreover, environmental distance was more important than was geographic distance for phylogenetic and functional β diversity between paired elevational bands. These results support the hypothesis that niche-based deterministic processes such as environmental filtering and competitive exclusion are fundamental in structuring woody plant assemblages along temperate elevational gradients regardless of scale (regional vs. local) in our study areas.

## Introduction

Biodiversity is defined as the variety of life forms at all levels of biological organization, including taxonomic, genetic, phenotypic, phylogenetic and functional diversity^1^. For biodiversity conservation and sustainability, understanding and investigating the patterns and drivers of biodiversity along environmental gradients are essential for determining biodiversity hotspots and management strategies in an ecosystem. In recent decades, species diversity was recognized and used as the most important component and measurement of biodiversity in several studies^2^. However, the definition of biodiversity contains three main components: species, functional and phylogenetic diversity. Recently, with expanded and advanced knowledge about phylogenetic relatedness and functional traits, phylogenetic and functional diversity and community structure analysis has become an important and popular new tool for differentiating the relative roles of niche-based deterministic (e.g., environmental filtering and species interaction) and neutrality-based stochastic (e.g., dispersal limitation and local extinction) processes that control plant community structure^3,4^. Therefore, ecologists have increasingly turned from species-centric approaches to phylogenetic and functional investigations to obtain more detailed information, such as the accumulated evolutionary and biogeographic history of communities in their study systems^3,5,6^. Recently, many ecological researchers have started to consider functional and phylogenetic diversity to complement the limitations and weaknesses of species diversity^2^ for various taxa, such as plants^2,6–8^, mammals^9^, birds^10^, insects^11^ and microorganisms^7^.

Mountain ecosystems provide a promising and well-organized natural laboratory for studies on biodiversity because the elevational gradients that have formed on mountains control the ecological and physiological adaptations of various organisms, such as plants, mammals, birds and invertebrates. Therefore, these gradients are recognized as the most important physical factors determining biodiversity and species distribution patterns on mountains^12^. Furthermore, the elevational gradient has often been found to be parallel to the latitudinal gradient^13^. Studies on elevational diversity patterns have been popular research subjects in ecology and biogeography for two decades, and there is extensive evidence for the patterns of species diversity and their underlying mechanisms^14–17^. However, to date, studies related to the other two biodiversity facets, i.e., phylogenetic and functional diversity, have been very rare. Moreover, most of the mechanisms that have been proposed to explain the relationship between diversity and elevation aim to clarify broad large-scale patterns and do not fully explain the elevational diversity patterns observed at smaller scales, such as local slope^18,19^. However, diversity patterns can change with spatial grain and scale^15^, and there is clearly a need to explore such small-scale patterns^20^. The lack of such analysis is partly due to constraints, such as the nature of data sets and the methodologies commonly used in macroecology, especially the dependence on secondary distribution data from the literature as well as the large number of proposed mechanisms^18^. Interest in studies on multifaceted approaches to biodiversity can be expanded by partitioning biodiversity into α, β and γ diversity^21,22^. α and γ diversity relate to the diversity patterns at a single site or in a specific habitat, sharing the same characteristics and differentiated only by scale. The complementary use of β diversity reflects the turnover among communities. Although this decomposition of biodiversity facets into α, β and γ components has been shown to be valuable in biodiversity conservation and management, it remains unclear whether these complementary components have similar trends and underlying mechanisms along the same environmental gradients.

We investigated the phylogenetic and functional diversity patterns of woody plant assemblages along one regional and two local elevational transects in temperate forests of South Korea. We also evaluated the ability of specific variables to explain these diversity patterns. Using data collected in field surveys, we investigated 1) the patterns of phylogenetic and functional α, β and γ diversity along temperate elevational gradients, 2) whether the patterns are different between regional and local transects or even between two local transects with different peaks in elevation, and 3) which environmental variables or distance matrices (environmental or geographic) play more important roles in shaping these diversity patterns.

## Results

### Phylogenetic signal

Regarding phylogenetic signal, Blomberg’s *K* and Pagel’s *λ* values for each of the functional traits were less than 1, with the values of Blomberg’s *K* being lower than those of Pagel’s *λ* (Table 1). However, all of the traits exhibited significant phylogenetic signal except for flowering onset according to Blomberg’s *K*. The results suggest that using phylogenetic distance as a proxy for differences in functional traits is appropriate for woody plant species in this study.

**Table 1:** Results of tests of phylogenetic signal in the functional trait data from three elevational transects using Blomberg’s *K* and Pagel’s *λ* statistics. *P < 0.05; **P < 0.01; ***P < 0.001.

Functional trait	Blomberg’s K	Pagel’s λ
Tree height (m)	0.199^***^	0.856^***^
Leaf length (cm)	0.060^*^	0.764^***^
Leaf width (cm)	0.193^***^	0.815^***^
Flowering onset (month)	0.029	0.672^***^
Seed weight (mg)	0.283^***^	0.984^***^

### Phylogenetic and functional diversity with elevation

**Figure 1:**
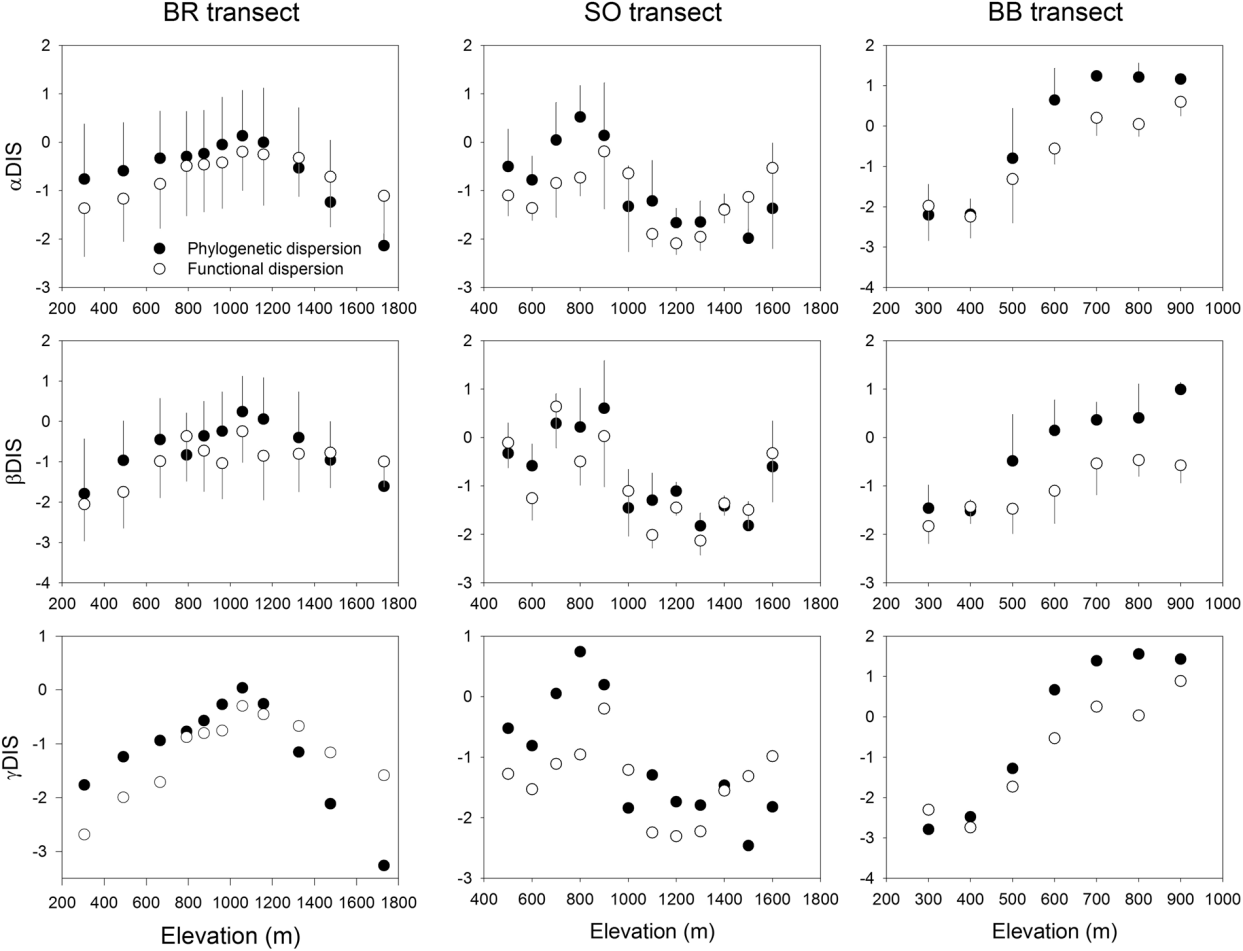
Relationships between elevation and the three components (α, β and γ) of phylogenetic and functional diversity along the three study transects. Half lines on black and white circles in α and β components indicate standard deviations. DIS indicates phylogenetic or functional dispersion.

**Figure 2:**
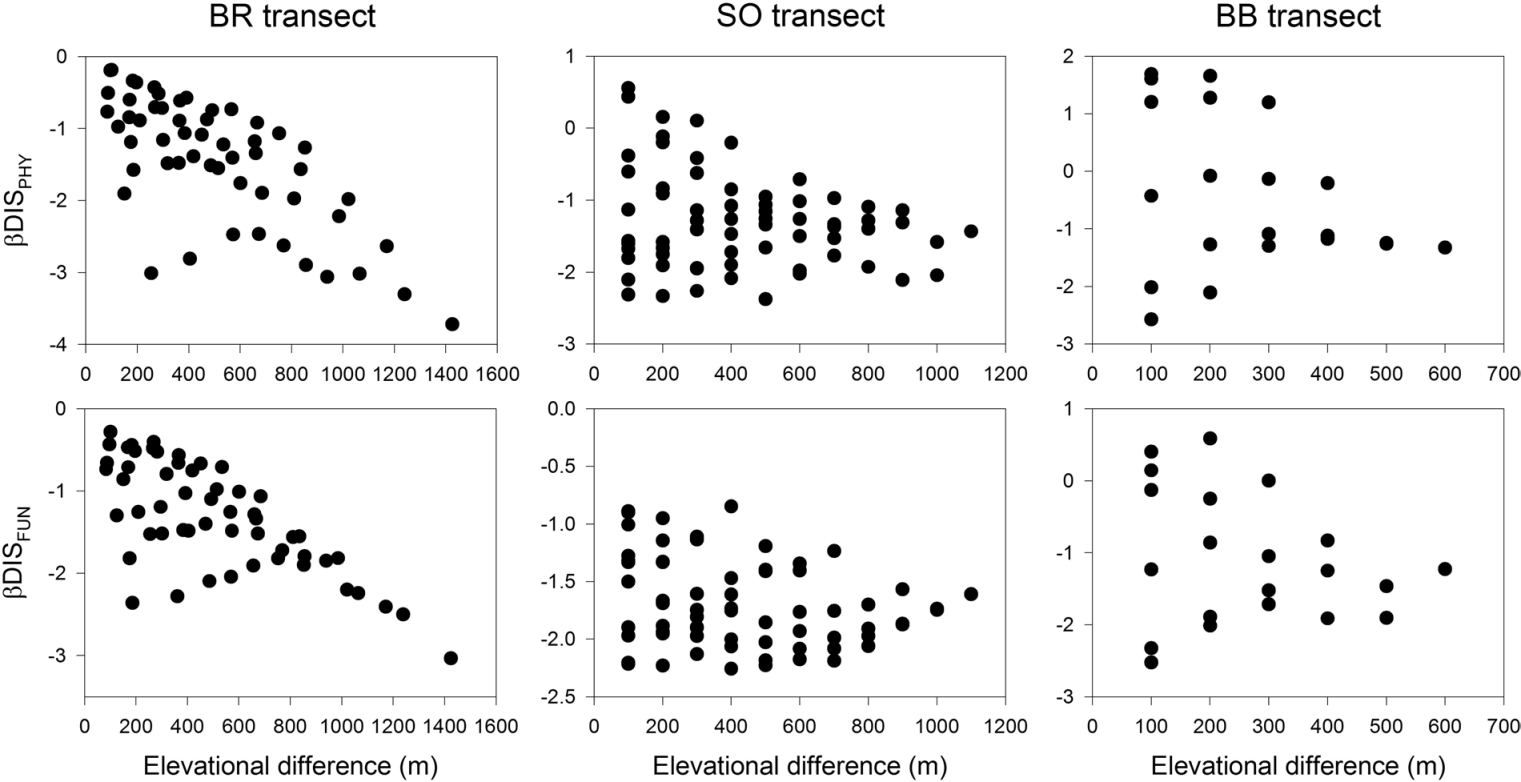
Relationships between elevational difference and the β components of phylogenetic and functional diversity between paired elevational bands in the three study transects. The correlation coefficients and significance levels from simple Mantel tests are shown in Table 3. DIS_PHY_ and DIS_FUN_ indicate phylogenetic and functional dispersion, respectively.

The three components of phylogenetic and functional diversity, which were derived for elevational bands (Table S1) in each study transect (Fig. S1; Table S2), exhibited significant quadratic relationships with elevation in the Baekdudaegan ridge (BR) transect (Fig. 1; Table S3). Whereas the phylogenetic diversity components were negatively correlated with elevation in the Osaek transect on Mt. Seorak (SO transect), the functional diversity components had no relationship with elevation. Furthermore, the three components of phylogenetic and functional diversity in the Bohyunsa transect on Mt. Baekhwa (BB transect) exhibited significant linear and quadratic relationships with elevation. However, the linear patterns were considered to provide better fit in the BB transect because the linear relationships had lower Akaike information criterion values than did the quadratic models (Table S3). The β components of phylogenetic and functional diversity between paired elevational bands had significant negative correlations with elevational differences except for functional β diversity in the BB transect (Fig. 2).

### Drivers of phylogenetic and functional diversity

The results of the simple ordinary least squares (OLS) regressions indicated that the α and γ components of phylogenetic diversity were related to regional area (RArea) and that the β component was correlated with habitat heterogeneity, whereas the functional diversity components were correlated only with habitat heterogeneity in the BR transect (Table 2). In the SO transect, the functional diversity components were related to habitat heterogeneity, whereas the phylogenetic diversity components were mainly correlated with climatic variables. In the BB transect, the phylogenetic diversity components were correlated with climatic variables. However, the functional diversity components were related to RArea, climatic variables and habitat heterogeneity. Analysis of the relationships of elevational difference or environmental distance matrices with β components between paired elevational bands using simple Mantel tests revealed that although the relative importance of the distance matrices differed among diversity indices and study transects, overall, environmental distances were more important than was elevational difference as a surrogate of geographic distance (Table 3). The results of the Mantel tests also indicated that the relative importance of elevational difference was higher in the regional (BR) transect than in the two local (SO and BB) transects.

**Table 2:** Coefficient of determination (*R*^2^) and significance level from simple ordinary least squares regression models for environmental variables and the three components (α, β and γ) of phylogenetic and functional diversity derived in elevational bands along the three study transects. DIS_PHY_ and DIS_FUN_ indicate phylogenetic and functional dispersion, respectively. *P < 0.05; **P < 0.01; ***P < 0.001.

Transect	Diversity index	RArea	PC1_clim_	PC1_hetero_	PC2_hetero_
BR	αDIS_PHY_	0.787^***^	0.294	0.195	0.004
	βDIS_PHY_	0.269	0.002	0.567^**^	0.396^*^
	γDIS_PHY_	0.704^***^	0.191	0.291	0.031
	αDIS_FUN_	0.073	0.079	0.549^**^	0.567^**^
	βDIS_FUN_	<0.001	0.209	0.508^*^	0.583^**^
	γDIS_FUN_	0.013	0.192	0.592^**^	0.694^***^
SO	αDIS_PHY_	0.398^*^	0.549^**^	0.195	0.245
	βDIS_PHY_	0.211	0.404^*^	0.273	0.222
	γDIS_PHY_	0.442^*^	0.568^**^	0.127	0.215
	αDIS_FUN_	<0.001	0.029	0.505^**^	0.174
	βDIS_FUN_	0.037	0.240	0.678^***^	0.099
	γDIS_FUN_	0.003	0.052	0.499^**^	0.195
BB	αDIS_PHY_	0.488	0.868^**^	0.473	0.142
	βDIS_PHY_	0.687^*^	0.904^***^	0.561	0.120
	γDIS_PHY_	0.514	0.888^***^	0.515	0.147
	αDIS_FUN_	0.645^*^	0.832^**^	0.630^*^	0.229
	βDIS_FUN_	0.516	0.821^**^	0.670^*^	0.175
	γDIS_FUN_	0.666^*^	0.818^**^	0.642^*^	0.244

Abbreviations: BR – Baekdudaegan ridge transect; SO – Osaek transect in Mt. Seorak; BB – Bohyunsa transect in Mt. Baekhwa; RArea – regional area with log-transformation; PC1_clim_ – PC1 from climatic variables; PC1_hetero_ – PC1 from standard deviations of topographic variables; PC2_hetero_ – PC2 from standard deviations of topographic variables.

**Table 3:** Results of simple Mantel tests to investigate the effects of elevational and environmental distances on phylogenetic and functional β diversity derived between paired elevational bands along the three study transects.

Transect	Diversity index	*Dist*_*ele*_	*Dist*_*rarea*_	*Dist*_*clim*_	*Dist*_*habit*_
BR	βDIS_PHY_	−0.727^***^	−0.835^***^	−0.747^***^	0.057
	βDIS_FUN_	−0.716^***^	−0.408^***^	−0.635^***^	−0.306^***^
SO	βDIS_PHY_	−0.226^**^	−0.501^***^	−0.166	−0.127
	βDIS_FUN_	−0.241^**^	−0.108	−0.250^**^	−0.230^**^
BB	βDIS_PHY_	−0.311^*^	−0.121	−0.580^***^	−0.139
	βDIS_FUN_	−0.226	0.228	−0.449^**^	−0.079

DIS_PHY_ and DIS_FUN_ indicate phylogenetic and functional dispersion, respectively. *P < 0.05; **P < 0.01; ***P < 0.001 Abbreviations: *Dist*_*ele*_ – elevational difference; *Dist*_*rarea*_ – regional area distance; *Dist*_*clim*_ – climate distance; *Dist*_*habit*_ – habitat heterogeneity distance. The abbreviations for the study transects are defined in Table 2.

**Figure 3:**
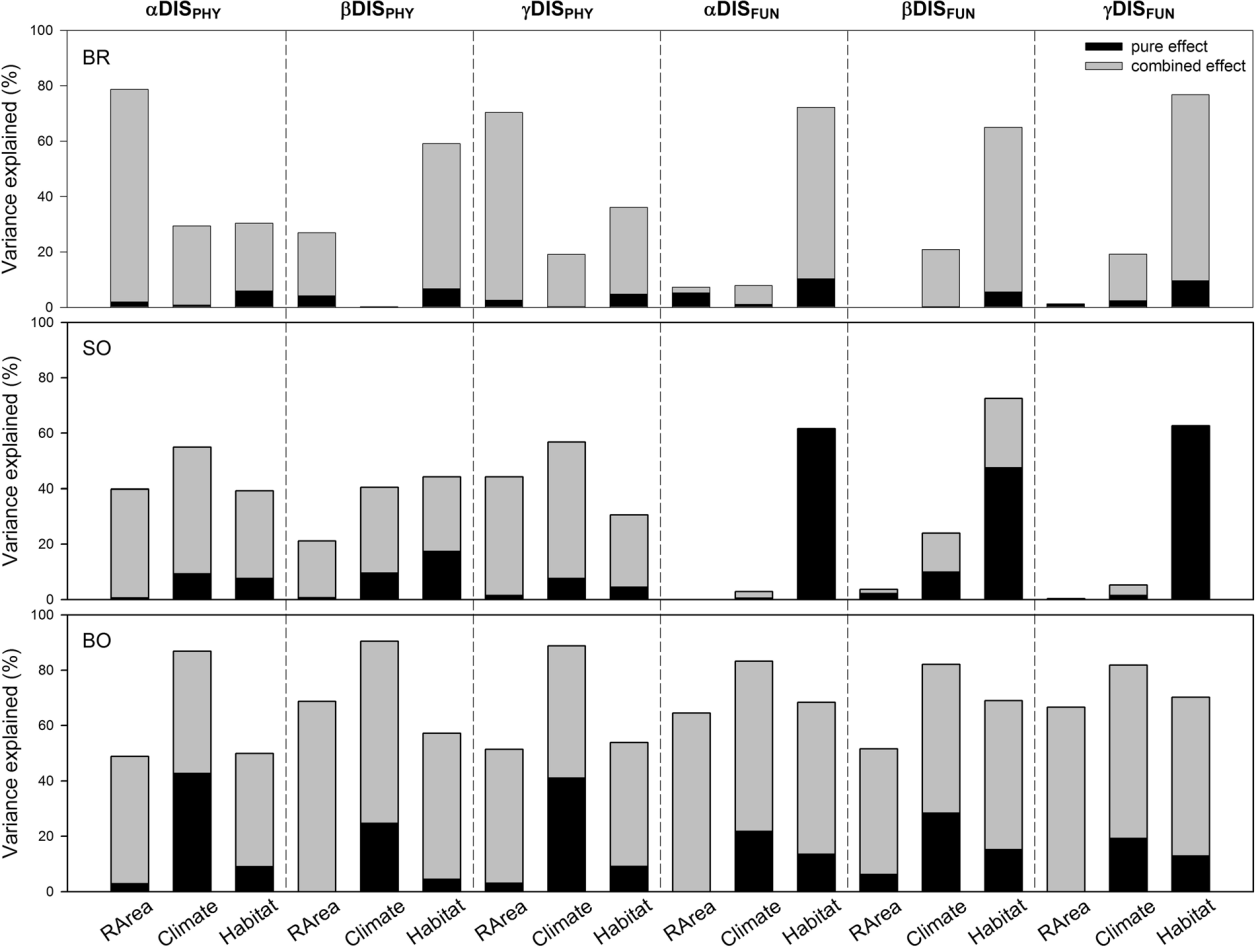
Variation partitioning of the three components (α, β and γ) of phylogenetic and functional diversity derived in elevational bands explained by regional area, climate and habitat heterogeneity along the three study transects. The black bars indicate the individual effects and the grey bars indicate the combined effects. Because the variance in αDIS_FUN_ and βDIS_FUN_ explained by regional area along the SO and BR transects, respectively, is very low (0.1%), the effects are not shown in this figure. The abbreviations for the study transects, explanatory variables and diversity indices are defined in Table 2. DIS_PHY_ and DIS_FUN_ indicate phylogenetic and functional dispersion, respectively.

**Figure 4:**
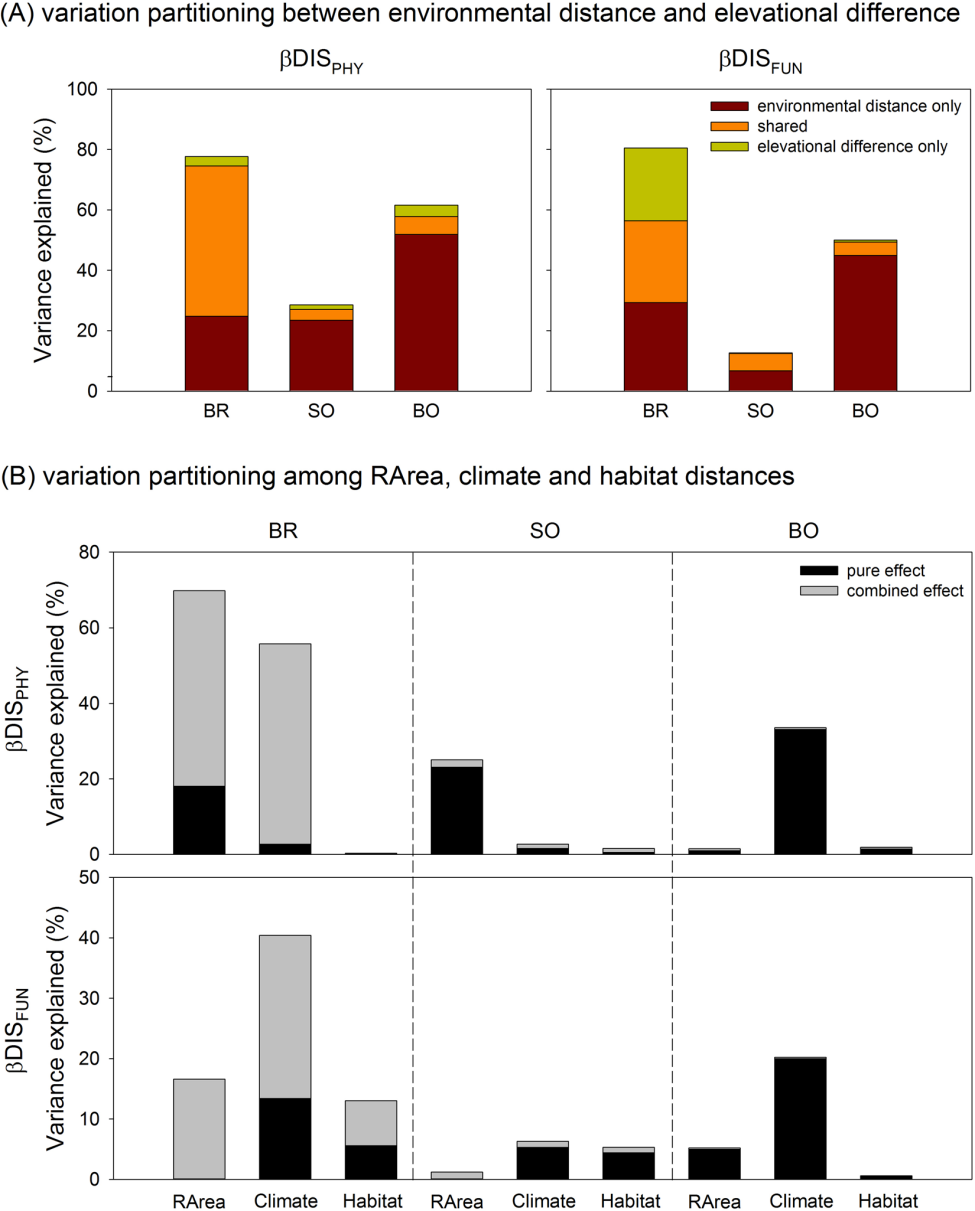
Variation partitioning of the β diversity components derived between paired elevational bands explained by (**A**) geographic and environmental distances and (**B**) each environmental distance, including RArea, climate and habitat distances along the three study transects. The abbreviations for the study transects, explanatory variables and diversity indices are defined in Tables 2 and 3. DIS_PHY_ and DIS_FUN_ indicate phylogenetic and functional dispersion, respectively.

The results of stepwise multiple regression models were similar to those of the simple OLS regression and the simple Mantel tests (Table 4). In the BR transect, RArea and habitat heterogeneity were the most important factors affecting the phylogenetic diversity components derived in the elevational bands, whereas climatic variables were the most import factors in the SO and BB transects. Habitat heterogeneity was more important for the functional diversity components except the β component in the BB transect. Furthermore, the environmental distances were more important factors than was elevational difference for phylogenetic and functional β diversity between paired elevational bands in the study transects. RArea and climatic distance were important factors affecting phylogenetic and functional β diversity between paired elevational bands. The simple conditional autoregressive (CAR) models showed similar results to those of the simple OLS models (Table S4), and the results of multiple CAR models supported the results of the best models resulted from forward stepwise multiple regression models (Table S5). The results of the variation partitioning also largely reinforced the results of the stepwise multiple regression models (Figs 3 and 4).

**Table 4:** Results of forward stepwise multiple regression models of the explanatory variables and the three components (α, β and γ) of phylogenetic and functional diversity along the study transects.

	Study transect	Dependent variable	Regression equation	*F*	*R* ^*2*^
Within elevations	BR	αDIS_PHY_	y = −5.036 + 1.064 RArea	33.203	0.787^***^
		βDIS_PHY_	y = −1.845 + 1.009 PC1_hetero_	11.781	0.567^**^
		γDIS_PHY_	y = −7.389 + 1.488 RArea	21.398	0.704^***^
		αDIS_FUN_	y = −1.216 − 2.111 PC2_hetero_	11.798	0.567^**^
		βDIS_FUN_	y = −1.692 − 2.817 PC2_hetero_	12.588	0.583^**^
		γDIS_FUN_	y = −2.285 − 4.256 PC2_hetero_	20.433	0.694^***^
	SO	αDIS_PHY_	y = −0.450 + 0.281 PC1_clim_	12.167	0.549^**^
		βDIS_PHY_	y = −0.348 + 0.250 PC1_clim_	6.772	0.404^*^
		γDIS_PHY_	y = −0.465 + 0.350 PC1_clim_	13.139	0.568^**^
		αDIS_FUN_	y = −0.985 + 0.261 PC1_hetero_	10.194	0.505^**^
		βDIS_FUN_	y = −0.638 + 0.431 PC1_hetero_	21.023	0.678^***^
		γDIS_FUN_	y = −1.234 + 0.266 PC1_hetero_	9.952	0.499^**^
	BB	αDIS_PHY_	y = 4.717 − 2.084 PC1_clim_	32.764	0.868^**^
		βDIS_PHY_	y = 2.808 − 1.302 PC1_clim_	47.121	0.904^***^
		γDIS_PHY_	y = 5.749 − 2.563 PC1_clim_	39.823	0.888^***^
		αDIS_FUN_	y = 2.542 − 1.364 PC1_clim_ − 0.323 PC2_hetero_	47.417	0.960^*^
		βDIS_FUN_	y = 0.567 − 0.699 PC1_clim_	23.002	0.821^**^
		γDIS_FUN_	y = 3.186 − 1.679 PC1_clim_ − 0.421 PC2_hetero_	45.861	0.958^*^
Between elevations	BR	βDIS_PHY_	y = − 0.381 − 1.083 *Dist*_*rarea*_ − 0.001 *Dist*_*ele*_	80.880	0.757^***^
		βDIS_FUN_	y = − 0.605 − 0.905 *Dist*_*clim*_ + 0.006 *Dist*_*ele*_	50.982	0.662^***^
	SO	βDIS_PHY_	y = − 0.896 − 0.705 *Dist*_*rarea*_	21.461	0.251^***^
		βDIS_FUN_	y = − 1.363 − 0.063 *Dist*_*clim*_ − 0.078 *Dist*_*habit*_	4.506	0.125^*^
	BB	βDIS_PHY_	y = 0.473 + 1.011 *Dist*_*rarea*_ − 2.118 *Dist*_*clim*_	11.638	0.564^***^
		βDIS_FUN_	y = − 0.700 + 0.755 *Dist*_*rarea*_ − 1.264 *Dist*_*clim*_	8.270	0.479^**^

The abbreviations for the study transects, explanatory variables and diversity indices are defined in Tables 2 and 3. *P < 0.05; **P < 0.01; ***P < 0.001.

## Discussion

In this study, we explored regional and local elevational patterns of the α, β and γ components of phylogenetic and functional diversity in woody plant assemblages and their associated drivers using primary data along different transects on different mountains. Thus, the present study is different from many other studies that examined broad large-scale trends using secondary data from literature reviews^14,15^. Moreover, this study investigated whether significant phylogenetic signal was present in the functional trait data to better understand the degree to which the phylogenetic tree can estimate the functional trait similarity of species. The study of biodiversity patterns at the regional (large) scale is critical for understanding the patterns across spatial scales, whereas the study of biodiversity patterns at the local (small) scale is salient for understanding the within-domain biodiversity in biogeographic groups^18,23^. This study provides a valuable contribution by exploring elevational patterns and the underlying mechanisms using empirical data collected simultaneously at regional and local scales.

### Phylogenetic signal

We found low levels of phylogenetic signal for the functional traits (i.e., Blomberg’s *K* and Pagel’s *λ* values < 1). Low phylogenetic signal is frequently interpreted as evolutionary trait lability or high rates of trait evolution contributing to large differences among close relatives. In this study, we quantified phylogenetic signal for five woody functional traits, and with the exception of flowering onset as based on Blomberg’s *K*, all of these traits exhibited significant phylogenetic signal. The general congruence of functional and phylogenetic dispersion is supported by significant phylogenetic signal in the trait data, but the congruence does not show ‘perfect correlation’ between the two dispersion patterns in our study transects (Fig. S2). This result can be understood by noting that the *K* and *λ* values of phylogenetic signal were all less than 1, suggesting that functional traits are more unstable than expected under a Brownian motion model of trait evolution. Previous studies have reported similar results in other forests, with phylogenetic and functional dispersions found to be aligned imperfectly but significantly with phylogenetic signal in trait data^24–26^. Thus, our results suggest that if there are significant but low values of phylogenetic signal in functional traits, phylogenetic dispersion can provide a rough approximation of functional dispersion; however, the correlations between both facets of diversity are not perfect. Moreover, if there is phylogenetic clustering in woody plant assemblages along elevational gradients in our study areas, our results might suggest the possibility of a direct connection between phylogenetic clustering and environmental filtering as one niche-based deterministic process structuring community assembly. The notion that phylogenetic clustering is mainly derived from strong environmental filtering rather than competitive exclusion relies on the assumption that the functional traits that are involved in community assembly processes have detectable phylogenetic signal^27,28^. Accordingly, ecologists and biogeographers increasingly recognize that testing for phylogenetic signal in functional traits is a necessary step when implementing phylogenetic community structure analysis^5,27,28^.

However, our results also raise the question of why these ‘not perfect correlations’ occur between phylogenetic relatedness and functional traits. First, phylogenetic relatedness is generally used as an indirect estimate of ecological similarity^29^. Therefore, phylogenetic relatedness serves as only an indirect proxy of the overall trait similarity of species in a community and thus may be unable to reveal similarities in individual functional traits. Second, although the estimation of general similarity for species is useful and tractable in some circumstances, this estimation is likely to overlook important information relevant to one or a few traits of species. Thus, much information can be lost when using phylogenetic relatedness as a proxy for trait similarity^2,6^. Moreover, an additional problem is that community assembly and species coexistence might be primarily influenced by a single resource axis, and only one functional trait might be important for understanding the processes underlying community assembly; however, phylogenetic relatedness likely cannot detect such processes^29^. However, these limitations similarly apply when using functional traits as a substitute for direct ecological similarity. Many studies relevant to the functional trait approach, including our study, use a few, easily measurable indirect traits and fundamental aspects of ecological strategy and functional trade-offs (e.g., morphological and structural traits, nutrient content) related to physiological processes in plants^2,8^. This approach is used because it is impossible to measure all traits thought to be important for physiological and defence mechanisms. Moreover, we do not know which traits are important for evolutionary processes in community assembly, and the functional trait approach also has inherent weaknesses such as the presence of intra- and inter-specific variation^2,8,29^. These limitations and shortcomings of both phylogenetic relatedness and functional trait approaches are likely to result in imperfect correlations between both phylogenetic and functional dispersions. Therefore, our results re-emphasize that studies on the structure of community assembly and the underlying processes should use both approaches complementarily, as emphasized in previous studies^2^.

### Diversity patterns and drivers along elevational gradients

The α, β and γ components of phylogenetic and functional diversity derived from the elevational bands in each transect showed different patterns, including hump-shaped curves, decreasing trends, increasing trends, and no relationship, with increasing elevation across the study transects. At the regional scale, i.e., in the BR transect, the three components of phylogenetic and functional diversity showed increasing phylogenetic and functional relatedness up to intermediate elevations (1000–1100 m), and then decreased thereafter. The patterns of phylogenetic and functional diversity were different between two local transects. Along the SO transect, decreasing phylogenetic relatedness (i.e., phylogenetic overdispersion) and no relationship of functional relatedness (i.e., functional randomness) with increasing elevation were observed, whereas along the BB transection, both phylogenetic and functional relatedness increased with increasing elevation, indicating phylogenetic and functional clustering. Moreover, as drivers shaping these patterns, RArea and the climatic variables were the most important factors influencing phylogenetic diversity along the regional- (BR) and local-scale (SO and BB) transects, respectively. In addition, habitat heterogeneity, based on topographic characteristics, was the main driver shaping the patterns of functional diversity components, which were derived in elevational bands.

The hump-shaped pattern of phylogenetic diversity with increasing elevation along the BR transect differs from the findings of previous studies reporting phylogenetic clustering or overdispersion of plant communities along elevational gradients^10,30–32^. RArea for the α and γ components and habitat heterogeneity for the β components were the most important factors. These results indicate that mid-elevational bands of larger area or higher habitat heterogeneity along the BR transect might exhibit increased phylogenetic relatedness with stable species colonization and extinction rates, whereas lower or higher elevational bands of smaller area or lower habitat heterogeneity might show more phylogenetically overdispersed patterns, with rapid, repeated colonization and extinction^33^. The ecological processes governing phylogenetic community structure differ among spatial scales. It is generally recognized that small-scale dispersal and species interactions are more important at local scales and that colonization and extinctions are crucial factors at regional scales^5,27,28^.

There are several potential explanations for the increase in phylogenetic overdispersion with increasing elevation in the SO transect. One potential explanation involves competitive exclusion: If competitive exclusion primarily removes related species of ecological similarity that show strong niche overlap at high elevations and if the degree to which different species have traits that favour them in competition for limited resources at high elevations is positively related to phylogenetic distance, then competition will drive phylogenetic overdispersion^34,35^. An alternative plausible explanation involves climatic variables, such as temperature differences between the hottest and coldest months along the SO transect. The temperature difference between the hottest and coldest months is generally recognized as temperature seasonality or variability. In the SO transect, temperature difference increased with increasing elevation, whereas a declining pattern was observed in the BR and BB transects (Fig. S3). This wide temperature range at higher elevations along the SO transect might allow some phylogenetically distant species that adapted to such cold conditions and wide temperature ranges during their evolutionary history to be distributed here to a greater extent than are closely related species. Accordingly, several genera such as *Lonicera*, *Syringa*, *Thuja*, and *Hydrangea* are common at high elevations (>1100 m) along the SO transect (Table S6). These genera represent the contribution of completely novel lineages to the woody plant assemblages at higher elevations along this transect^23^, and many other genera are widely distributed across the entire elevation range along the SO transect (Table S6). These potential explanations are not mutually exclusive, and thus one or more might be involved in contributing to the phylogenetic overdispersion observed at higher elevations along the SO transect.

The clustering patterns of the phylogenetic diversity components along the BB transect may be explained by a stronger effect of environmental filtering than of competitive exclusion^5,7^ in shaping community assembly at high elevations. In particular, climate-related filtering, in which closely related species with adaptive traits to harsh and stressful environmental conditions, such as low temperature and strong wind, are filtered^34,36,37^, might play a strong role. The lower phylogenetic relatedness at low elevations can be interpreted as evidence that the effect of competitive exclusion among related species with ecological similarity is stronger than the environmental filtering effect such that these clades are distantly related to other temperate lineages at low elevations^27,28^. Accordingly, many genera were recorded at low elevations (<600 m) along the BB transect (11 genera, including *Juniperus*, *Smilax*, and *Zanthoxylum*), whereas a smaller number of genera (7 genera, including *Carpinus*, *Tripterygium*, and *Vitis*) were observed at high elevations (>600 m) (Table S6). This difference in the number of unique genera between low and high elevations represents a phylogenetic clustering pattern along the BB transect. Generally, in most previous studies on elevational patterns of phylogenetic diversity, phylogenetic clustering at higher elevations has been observed in response to abiotic filtering, such as environmental filtering^10,26,30,34,36^, whereas few studies have revealed phylogenetic overdispersion in response to biotic interactions, such as interspecific competition, at higher elevations^31,32^. Although the patterns of phylogenetic diversity and the underlying processes along elevational gradients differed among the study transects in this study and among studies, the underlying mechanisms mainly involve niche-based deterministic processes, including abiotic (e.g., environmental filtering by climate gradients) and biotic (e.g., competition) processes^36^. The most influential environmental factor in shaping phylogenetic diversity patterns in the two local-scale transects was the climatic factor. This finding highlights climatic variables as one of the main drivers of biodiversity^36,37^, which supports concerns regarding the effects of climate on species distribution and composition in many areas and on related ecosystem services and evolutionary responses^38,39^. Our results also indicate that the relative importance of different environmental factors on phylogenetic diversity derived in elevational bands might be scale dependent.

The functional diversity components exhibited patterns similar to those of the phylogenetic diversity components. Although the α, β and γ components of functional diversity along the SO transect did not show significant linear or quadratic relationships with elevation, suggesting random functional structure along the SO transect, we cannot infer a role of neutrality-based stochastic processes in this structure because the pattern was closely related to the gradient of habitat heterogeneity, as represented by PC1_hetero_. Therefore, the processes shaping the patterns of the functional diversity components were related to the gradients of climate and habitat heterogeneity along the BB transect and mainly to the gradients of habitat heterogeneity along the other transects. These results also support the hypothesis that niche-based deterministic processes are fundamental processes for functional diversity in woody plant assemblages along our study transects. Other studies have similarly documented the importance of climate^40^ and habitat factors^26,41^ in determining the functional structures of woody plant assemblages. Habitat heterogeneity is considered an important factor shaping diversity patterns, notably by driving local differences in species distribution and thereby increasing diversity via differences in environmental preferences^42^. Heterogeneity is also thought to significantly influence the dynamics and structure of ecological communities^43^. In particular, topographic heterogeneity has been recognized as creating a complex mosaic or heterogeneity of substrates and soils with varying structures, hydrology and chemistry^44^. In general, plant functional traits are strongly correlated with soil resources and water availability at the community scale^19^. Therefore, spatial heterogeneity in nutrient and water availabilities, along with other environmental factors derived from topographic heterogeneity, could influence the functional diversity of woody plants that coexist via niche partitioning^45,46^. In heterogeneous habitats, heterogeneous resources, including light, moisture and soil nutrients, allow plant species with different niche requirements to meet their habitat requirements, which leads to higher functional diversity^47^. Therefore, our results indicate that woody species distributions along topographic gradients may be partly shaped by habitat filtering through a selection of functional traits that are associated with tree resource use and growth strategies^48^.

Although our study emphasizes the importance of niche-based deterministic processes for structuring the patterns of phylogenetic and functional diversity in elevational bands, as evidenced in the different study transects, the implications of these processes for phylogenetic and functional diversity appear to differ. Specifically, the processes associated with phylogenetic diversity patterns varied among the study transects and the three components, whereas the processes structuring functional diversity were mainly associated with the gradients of habitat heterogeneity in the study transects. These results suggest that phylogenetic diversity, which reflects the accumulated evolutionary and biogeographic history of community assembly, is potentially associated with various abiotic and biotic processes, whereas functional diversity, which is related to ongoing ecological processes as inferred from morphological, physiological and ecological traits, is associated with a set of species with functional traits that are optimally adaptable for a given environment or set of habitat conditions regardless of the phylogenetic distance among lineages.

With the exception of functional β turnover along the BB transect, the phylogenetic and functional β turnovers between paired elevational bands along all of the study transects indicated significant linear decreases. Such patterns have been observed in many previous studies and are collectively well known as the distance-decay relationship, which describes the decrease in compositional similarity between two communities with increasing geographic distance (or equivalent elevational difference) between them^49^. The β components of phylogenetic and functional dispersions between paired elevational bands in the present study were mainly governed by regional area and climatic distances, although elevational difference as a proxy for geographic distance was also important in structuring the β components along the regional transect (BR). Our results indicate that environmental distance is generally a better predictor of β diversity than is geographic distance, thereby suggesting more support for deterministic processes than for stochastic processes^2^. Moreover, our study supports previous findings that phylogenetic or functional turnover is significantly related to environmental gradients from local to regional scales^50^.

In summary, we observed low but significant phylogenetic signal in functional traits, suggesting that phylogenetic dispersion can roughly approximate functional dispersion but that the two facets of diversity are not perfectly correlated. Although the patterns of phylogenetic and functional diversity differed among temperate elevational gradients at both regional and local scales in the study areas in South Korea, the main drivers were partitioned into two categories: regional area and climatic variables for phylogenetic diversity and habitat heterogeneity for functional diversity in elevational bands. Furthermore, environmental distance was a more important predictor of β components between paired elevational bands than was geographic distance. Our study generally supports the hypothesis that niche-based deterministic processes, such as biotic (e.g., competitive exclusion) and abiotic (e.g., environmental filtering by habitat and climatic factors) processes, are fundamental mechanisms structuring woody plant assemblages along temperate elevational gradients in South Korea. Moreover, the results suggest that the environmental drivers might differ between the considered facets of diversity and among scales.

## Methods

### Study area and plant data

To evaluate the differences in phylogenetic and functional diversity patterns and the relationships of the diversity patterns with explanatory variables along regional and local elevational transects, we selected the main ridge of the Baekdudaegan Mountains as a regional (BR) transect and two local (SO and BB) transects, one on Mt. Seorak and one on Mt. Baekhwa (Fig. S1; Table S2). A total of 256 woody plant species representing 50 families and 101 genera were recorded from 1195 400-m^2^ (20 m × 20 m) forest plots along the three transects during the growing season (May to August) of 2005 to 2011 (Table S2). Detailed descriptions of the study area and plant data are described in Supplementary Methods.

### Phylogenetic tree and functional trait dendrogram

A phylogenetic tree of the woody plant species surveyed in this study was generated by pruning the PhytoPhylo megaphylogeny^51^, which is an updated version of the vascular plant phylogeny published by Zanne *et al*.^52^. This megaphylogeny is the largest phylogeny of vascular plants available and was generated based on the sequences of seven gene regions (i.e., 18S rDNA, 26S rDNA, ITS, *mat*K, *rbc*L, *atp*B and *trn*L-F) from GenBank. The phylogeny includes all families of extant seed plants in the world and was time-scaled based on 39 fossil calibrations. The 101 genera surveyed in our study were present in PhytoPhylo. The S.PhyloMaker function with Scenario 3 was implemented in R software to assign those species that were not present in PhytoPhylo^52^.

To construct a functional trait dendrogram for quantifying functional diversity, we included five functional traits for all woody plant species: maximum height (*m*), leaf dimensions (*cm*; length and width), flowering onset (*month*) and seed mass (*mg*). Values of all traits were log transformed to improve normality and were standardized before analysis. To eliminate trait redundancy, we performed a principal component analysis (PCA) on the functional trait data (Table S7). We used the first four principal components, which explained 94.8% of the variation in the trait data, to construct a Euclidean trait distance matrix. An unweighted paired group method with arithmetic mean (UPGMA) hierarchical clustering was then applied to this matrix to produce a trait dendrogram.

The phylogenetic tree and functional trait dendrogram (Fig. S4) were separately constructed for three scenarios. Additional information regarding the importance and data sources of the five functional traits and regarding the construction of the phylogenetic tree and trait dendrogram is provided in Supplementary Methods.

### Phylogenetic and functional dispersion

The abundance-weighted net relatedness index (NRI)^5^ was used to quantify the α and γ components of phylogenetic (or functional) dispersion. The formula is as follows:

1 $${\rm{NRI}}=\frac{-1\times ({{\rm{MPD}}}_{{\rm{observed}}}\,-{\mathrm{mean}\mathrm{MPD}}_{{\rm{random}}})}{{\mathrm{sd}\mathrm{MPD}}_{{\rm{random}}}}$$

where MPD_observed_ is the observed mean phylogenetic (or functional) distance (MPD) in each plot or elevational band, mean MPD_random_ is the mean MPD of the null models, and sd MPD_random_ is the standard deviation of MPD of the null models. MPD mainly reflects the deep phylogenetic (or functional) structure in a phylogeny. As such, MPD is typically thought to be more sensitive to tree-wide patterns of phylogenetic (or functional) clustering or overdispersion^5,24^ than to the structure near the tips.

To calculate phylogenetic (or functional) β dispersion between paired plots in each elevational band or paired elevational bands in each study transect, we quantified the standardized effect size (S.E.S.) of abundance-weighted D_pw_ (mean pairwise phylogenetic or functional distance) as follows:

2 $${\rm{S}}.{\rm{E}}.{\rm{S}}.\,{{\rm{D}}}_{{\rm{pw}}}=\frac{-1\times ({{\rm{D}}}_{{\rm{pw}}{\rm{observed}}}-{\rm{mean}}\,{{\rm{D}}}_{{\rm{pw}}{\rm{random}}})}{{\rm{sd}}\,{{\rm{D}}}_{{\rm{pw}}{\rm{random}}}}$$

3 $${{\rm{D}}}_{{\rm{pw}}}=\,\frac{{\sum }_{{\rm{i}}=1}^{{{\rm{nk}}}_{1}}{{\rm{f}}}_{{\rm{i}}}\overline{{{\rm{d}}}_{{{\rm{ik}}}_{2}}}+{\sum }_{{\rm{j}}=1}^{{{\rm{nk}}}_{2}}{{\rm{f}}}_{{\rm{j}}}\overline{{{\rm{d}}}_{{{\rm{jk}}}_{1}}}}{2}$$

where D_pw observed_ is the observed phylogenetic (or functional) dissimilarity between plots or elevational bands, mean D_pw random_ is the mean D_pw_ of the null models, and sd D_pw random_ is the standard deviation of D_pw_ of the null models. In the D_pw_ equation, $\bar{{d}_{{{ik}}_{2}}}$ is the mean pairwise phylogenetic (or functional) distance (MPPD) between species *i* in plot *k*_1_ or elevational band *k*_1_ to all species in plot *k*_2_ or elevational band *k*_2_, and $\bar{{d}_{{{jk}}_{1}}}$ is the MPPD between species *j* in plot *k*_2_ or elevational band k_2_ to all species in plot k_1_ or elevational band k_1_. *f*_*i*_ and *f*_*j*_ represent the relative abundances of species *i* and species *j*. This dissimilarity matrix is highly correlated with Rao’s *D* and is better than Rao’s *D* for detecting major compositional turnover between communities^8,24^.

To quantify phylogenetic (or functional) dispersion such as NRI and S.E.S. D_pw_, we created the null model by randomly shuffling the names of the species across the tips of the phylogenetic tree (or functional trait dendrogram) 1000 times. This approach randomized the phylogenetic (or functional trait) relatedness of the species to one another while maintaining the observed community data matrix. Therefore, this null model fixes the observed levels of species occupancy rates, abundances and spatial distributions in each randomization^2^.

Consequently, we calculated four indices, comprising one α component, two β components and one γ component, as subsets of phylogenetic (or functional) dispersion in this study. The α component was defined as the mean value of the NRIs of all plots in each elevational band. The β components were calculated in two ways: 1) as the mean values of S.E.S. D_pw_ between plots in each elevational band and 2) as the S.E.S. D_pw_ between paired elevational bands in each study transect using species data, which were pooled and summed from multiple plots in the same elevational band. Finally, the γ component was quantified in each elevational band of each transect. The COMSTRUCT function for the α and γ components and the COMDIST function for the β components were used in Phylocom 4.2^53^. We used the phylogenetic trees and trait dendrograms described in “*Phylogenetic tree and functional trait dendrogram*” to calculate the components of both dispersions.

One of the main aims of this study was to compare and contrast measurements of phylogenetic and functional diversity. Therefore, we quantified the degree of phylogenetic signal in each functional trait using Blomberg’s *K*^54^ and Pagel’s *λ*^55^ statistics to determine whether the phylogenetic tree can estimate the similarity of functional traits of species. Additional information related to the two statistics is provided in Supplementary Methods.

### Environmental variables

For the environmental variables, we included the RArea, three topographic heterogeneity variables and six climatic variables for each elevational band in the three transects. Detailed information on the calculation of the environmental variables is described in Supplementary Methods. The relationships between the environmental variables and elevation are shown in Fig. S5. Before performing further statistical analyses, RArea and two precipitation-related variables were subjected to log transformation to achieve normality. Furthermore, to reduce the co-variation and possible redundancy in the data, two separate PCAs were performed on each set of standard deviations of topographic variables and climatic variables (Table S8). From the PCA, the first PCA axis for the climatic variables and the first and second PCA axes for the topographic variables were extracted, creating new PCA-derived variables as independent variables. The PCA-derived variables were labelled PC1_clim_, PC1_hetero_ and PC2_hetero_ for climate and topographic heterogeneity.

To investigate the relationships between phylogenetic or functional β dispersion and geographic or environmental distance, we calculated the elevational difference as a proxy of geographic distance between elevational bands because elevational separation is generally recognized as correlating with geographic distance^49^. We also calculated the climatic distance with PC1_clim_ and the habitat distance with PC1_hetero_ and PC2_hetero_ using the Euclidean distance measurements, and the RArea distance was measured as the absolute value of the difference in RArea between paired elevational bands.

### Data analysis

Linear and quadratic regression models were fitted to assess the relationship between elevation and each of the three components of phylogenetic and functional diversity. To test the effects of individual variables, such as RArea, PC1_clim_, PC1_hetero_ and PC2_hetero_, on the elevation patterns of the three components of phylogenetic and functional diversity, we performed a simple OLS regression analysis. We also performed stepwise multiple regression to establish the relative importance of each environmental variable for the diversity indices. A simple Mantel test with 10000 permutations was applied to evaluate the significance of the correlation between the elevation or environmental distance matrix and each β component between the paired elevational bands. We also used simple and multiple CAR models to evaluate the influence of spatial autocorrelation, such as inflation of type I error and invalid parameter estimation, on the regression results^42,56^.

Studies focusing on mechanisms driving diversity patterns generally apply multiple regressions and similar statistical analyses. However, more complex strategies were applied here for the ecological data analyses because it is important to account for the lack of independence between pairs of observations across geographic space^57^. Therefore, in this study, we used variation partitioning^57^ with partial regressions for the α, β and γ components of phylogenetic and functional diversity with environmental variables, which were derived in elevational bands, and multiple regressions on distance matrices (MRM) for β components with distance matrices, which were derived between paired elevational bands.

## Electronic supplementary material

Supplementary Information

Supplementary Dataset 1 (Table S6)
