# Supplementary material for: Partitioning the regional and local drivers of phylogenetic and functional diversity along temperate elevational gradients on an East Asian peninsula
Source: Sci Rep. 2018 Feb 12;8:2853. doi: 10.1038/s41598-018-21266-4 (PMC5809509; doi:10.1038/s41598-018-21266-4)
Supplement: Supplementary file 1 — Supplementary Information [file 41598_2018_21266_MOESM1_ESM.doc]

**Supplementary Information**

**Partitioning the regional and local drivers of phylogenetic and functional diversity along temperate elevational gradients on an East Asian peninsula**

**Jung-Hwa Chun ^1^, Chang-Bae Lee ^2,*^**

*^1^ Division of Forest Ecology, National Institute of Forest Science, 57 Hoegiro, Dongdaemungu, Seoul 02455, Republic of Korea*

*^2^ Global Resources Division, Korea Forestry Promotion Institute, 475 Gonghangdaero, Gangseogu, Seoul 07570, Republic of Korea*

*Correspondence and requests for materials should be addressed to C.B.L (Email: cblee@kofpi.or.kr* Tel: +82-2-6393-2773 Fax: +82-2-6393-2609*)*

**Methods**

**Study area** The Baekdudaegan Mountains are the largest and most unique mountain range on the Korean peninsula and consist of approximately 487 mountains, hills and peaks, which are a major source of forest biodiversity^1^. The Baekdudaegan Mountains in South Korea belong to a mountain ecoregion and a temperate deciduous forest biome; the bedrock is composed of granite, granite gneiss and metamorphic sedimentary rocks^2^. The mean annual temperature ranges from a minimum of –5.87 °C in January to a maximum of 19.63 °C in August, and the mean annual precipitation varies between approximately 1200–2000 mm based on national digital climate maps produced by the National Center of AgroMeteorology at the Korean Meteorological Administration^3,4^. Mt. Seorak is the third-highest mountain in South Korea and was designated as the fifth Korean national park in 1970 and as a biosphere reserve in 1982 by the Korean government and UNESCO, respectively. Mt. Seorak has an area of 398.2 km^2^, with the highest peak on Daecheongbong (1708 m a.s.l.), and contains mountain ranges of dissected granite and gneiss^2^. The mean annual temperature and precipitation are 9.9 °C and 1114 mm, respectively^5^. Mt. Baekhwa has an area of 15.9 km^2^, with the highest peak on Hansungbong (933 m a.s.l.), and the bedrock is composed of granite and granite gneiss. The mean annual temperature and precipitation are 12.6 °C and 1259 mm, respectively^6^.

The vegetation on the Baekdudaegan Mountains can be divided into four major vegetation zones along an elevational gradient. These elevational vegetation zones include the following: (1) temperate deciduous broad-leaved and pine forests dominated by *Pinus densiflora* and *Rhus trichocarpa* (< 550 m a.s.l.); (2) temperate deciduous broad-leaved and coniferous mixed forest dominated by *Abies holophylla*, *P*. *koraiensis*, *Quercus* *mongolica* and *Q*. *serrata* (550–1100 m a.s.l.); (3) sub-alpine coniferous forests dominated by *Taxus* *cuspidata*, *A*. *koreana* and *A*. *nephrolepis* (1100–1600 m a.s.l.); and (4) dwarf sub-alpine forests dominated by *Betula ermanii* and *P*. *pumila* (> 1600 m a.s.l.)^2^. The vegetation on Mt Seorak is divided into four types along an elevational gradient as follows: (1) temperate (montane) deciduous and pine forest (< 500 m a.s.l.) dominated by *P. densiflora* and *Rhododendron mucronulatum* var. *mucronulatum*; (2) temperate deciduous and coniferous mixed forest (500–1100 m a.s.l.) dominated by *Q. mongolica*, *B*. *schmidtii*, *Magnolia sieboldii*, *P. koraiensis*, and *A. holophylla*; (3) subalpine coniferous forest (1100–1500 m a.s.l.) dominated by *T. cuspidata*, *Thuja koraiensis*, and *A. nephrolepis*; and (4) alpine forest (> 1500 m a.s.l.) dominated by *B. ermanii* and *P. pumila*^2^. Mt. Baekhwa is divided into three vegetation types with elevation as follows: (1) warm temperate deciduous forest (< 350 m a.s.l.) dominated by *Q. serrate* and *Platycarya strobilacea*, (2) temperate deciduous forest (350–600 m a.s.l.) dominated by *Zelkova serrata* and *R. schlippenbachii*, and (3) temperate deciduous broad-leaved and coniferous mixed forest (> 600 m a.s.l.) dominated by *P. densiflora* and *Acer pseudosieboldianum*^6^.

**Plant data** The species composition of 1195 forest plots of 400 m^2^ (20 m × 20 m) along 100-m-wide transects in three study areas in South Korea were recorded during the growing season (May to August) of 2005 to 2011 (Table S2). We studied 1100 plots, 60 plots and 35 plots along the BR, SO and BB transects, respectively. Although Mt. Seorak and Mt. Baekhwa are within the boundary of the Baekdudaegan Mountains, the two transects, SO and BB, did not overlap with the BR transect. Within each plot in the transects, the plant species and cover-abundance scale were exhaustively recorded for 1–2 h depending on the species richness of the plot in accordance with the methods of Braun-Blanquet^7^. A total of 256 woody plant species were recorded, representing 50 families and 101 genera. Supplementary Table S6 lists the woody plant species and their functional trait checklists at each elevational band along the three transects. The SO and BB transects were divided into 12 and 7 elevational bands, respectively, of 100-m intervals. However, BR was divided into 11 elevational bands of different elevation intervals but the same number of plots in each elevational band to eliminate the effect of sampling effort (Table S1).

**Phylogenetic tree and functional trait dendrogram** The five functional traits of woody plants, comprising maximum height, leaf length, leaf width, flowering onset and seed mass, considered in this study are believed to represent important aspects of plant strategy and fundamental functional trade-offs^8,9^. Maximum height is the dominant factor influencing access to light and represents a major axis of life history variation^10^. Leaf size has important consequences for leaf energy and water balance^8^. Seed mass is an indicator of dispersal and regeneration strategy and represents the trade-off between seed number and seed size^11^. Flowering onset is related to phenological and reproductive strategies and represents the trade-off between maximizing fruit set and reducing damage risk, especially in temperate forests^9^. We obtained trait values for all woody species in the dataset from the literature and a publicly accessible online database, as described in Table S6. When more than one value was available for a given species, a mean value was calculated, and each species in the dataset was assigned a mean trait value. Therefore, our study does not account for intra- and inter-specific trait variations.

The phylogenetic tree and functional trait dendrogram (Figure S4) were separately constructed for three scenarios and were used as follows. 1) Six phylogenetic trees and trait dendrograms for each transect were constructed to calculate the α and γ components of phylogenetic and functional dispersion in each plot and elevational band as well as the β components of both dispersion types between paired elevational bands. 2) Sixty phylogenetic trees and trait dendrograms for each elevational band in each transect were constructed to calculate the β components of both dispersion types between paired plots in each elevational band. 3) One phylogenetic tree containing all of the woody species from the three transects was constructed to quantify phylogenetic signal. The 34 phylogenetic trees and 33 trait trees were used to calculate phylogenetic signal and phylogenetic and functional dispersion.

**Phylogenetic signal** Our study aimed to compare and contrast measurements of phylogenetic and functional diversity. It is expected that the pattern of functional diversity should mirror the pattern of phylogenetic diversity if significant phylogenetic signal is present in the functional trait data. The degree of phylogenetic signal in the five functional traits was evaluated using Blomberg’s *K* and Pagel’s *λ* statistics. A Blomberg’s *K* value greater than 1 indicates stronger similarity among related species than expected under Brownian motion (BM), whereas a *K* value less than 1 indicates less similarity among related species than expected under BM. Pagel’s *λ* = 0 implies no phylogenetic signal, and *λ* = 1 indicates that the distribution of the trait is as expected under BM. To test the significance of the *K* and *λ* statistics, we randomly arrayed the trait data on the community phylogeny 1000 times to generate a null distribution.

**Environmental variables** To test the diversity-area relationships, we calculated the regional area (RArea) of each elevational band for the entire mountain range for each study mountain to estimate the habitat area that is available to support the regional species pool. Furthermore, three topographic heterogeneity variables, namely, the standard deviations of slope, the topographic position index^12^ and the surface area ratio^13^, were calculated to estimate the effects on plant diversity for each elevational band in the 100-m-wide transects. This was achieved by using a triangular irregular network model that was based on a digital elevation model developed from a 1:25000 digital terrain map. Calculations for RArea and the three topographic characteristics were performed using the digital elevation model with a resolution of 30 m as implemented with the 3D Analyst extension in ArcGIS. We also calculated the mean annual temperature, monthly mean temperature in the coldest month (January), monthly mean temperature in the hottest month (August), mean temperature in the growing season (May–August), and mean annual precipitation and mean precipitation in the growing season as climatic variables using national digital climate maps produced by the National Center of AgroMeteorology at the Korean Meteorological Administration^3,4^. The spatial resolutions of the raster data were 30 m and 270 m for the temperature- and precipitation-related variables, respectively. The temperature-related data were from the period 1971 to 2008, and the precipitation-related data were from the period 1981 to 2009.

**References**

1. Korea Forest Research Institute. *Ecological aspects of Baekdu Mountains in Korea and delineation of their management and conservation area*. (Korea Forest Research Institute, 2003).

2. Kong, W. S. *Biogeography of Korea plants*. (GeoBook Publishing, 2007).

3. Yun, J. I. Agroclimatic maps augmented by a GIS technology. *Korean J. Agric. For. Meteorol.* **12,** 63–73 (2010).

4. Chun, J. H. & Lee, C. B. Assessing the effects of climate change on the geographic distribution of Pinus densiflora in Korea using Ecological Niche Model. *Korean J. Agric. For. Meteorol.* **15,** 291–233 (2013).

5. Han, S. K. *et al*. Some unrecorded higher fungi of the Seoraksan and Odaesan National Parks. *Mycobiology* **34,** 56–60 (2006).

6. Cho, H. J., Lee, Y. W., Lee, D. S. & Hong, S. C. Forest vegetation of Mt. Baekhwa: a phytosociological study. *J. Korean For. Soc.* **80,** 42–53 (1991).

7. Braun-Blanquet, J. *Plant sociology: the study of plant communities*. (Hafner Publishing, 1965).

8. Cornelissen, J. H. C. *et al*. A handbook of protocols for standardized and easy measurement of plant functional traits worldwide. *Aust. J. Bot.* **51,** 335–380 (2003).

9. Garnier, E., Navas, M. L. & Grigulis, K. *Plant functional diversity: organism traits, community structure, and ecosystem properties*. (Oxford University Press, 2016).

10. Moles, A. T. *et al*. Global patterns in plant height. *J. Ecol.* **97,** 923–932 (2009).

11. Moles, A. T. & Westoby, M. Seed size and plant strategy across whole life cycle. *Oikos* **113,** 91–105 (2006).

12. Jenness, J. S. Topographic position index (tpi_jen.avx) extension for ArcView 3.x. (2006) Available at: http://www.jennessent.com/arcview/tpi.htm. (Accessed: 13th October 2016).

13. Jenness, J. S. Calculating landscape surface area from digital elevation models. *Wildl. Soc. Bull.* **32,** 829–839 (2004).

**List of Supplementary Figures**

**Figure S1.** Location and topography of the three study transects on (A) the Baekdudaegan Mountains, (B) Mt. Seorak and (C) Mt. Baekhwa in South Korea. All maps were created using ArcGIS version 9.3.1 (ESRI, Redlands, California, USA, <http://www.esri.com/>).

**Figure S2.** Relationships between components (α, β and γ) of phylogenetic and functional diversity along the three study transects. Pearson’s correlation analysis of the three components derived in elevational bands and Mantel tests of the β components derived between paired elevational bands were performed. The Mantel test was applied with 10000 permutations to evaluate the significance of each relationship.

**Figure S3.** Relationship between elevation and the difference in mean temperature between the hottest August and coldest January (h-cMT) along the three study transects. The abbreviations for the study transects are defined in Table S1.

**Figure S4.** Images of the representative phylogenetic trees and functional trait dendrograms used in this study; (A–B) total woody species, (C–D) woody species along the BR transect, (E–F) woody species along the SO transect and (G–H) woody species along the BB transect. This figure shows representative images for phylogenetic trees and functional trait dendrograms because we could not show all of the images of phylogenetic trees and functional trait dendrograms (i.e., 67 images). The abbreviations for the study transects are shown in Table S1.

**Figure S5.** Relationships between elevation and the explanatory variables evaluated in this study along the three study transects. The explanatory variables include regional area (RArea), mean annual temperature (MAT), mean temperature in the coldest month (cMAT), mean temperature in the hottest month (hMAT), mean temperature in the growing season (gMAT), mean annual precipitation (MAP), mean precipitation in the growing season (gMAP), standard deviation (SD) of the slope, SD of the topographic position index (TPI) and SD of the surface area ratio (SAR). The abbreviations for the study transects are defined in Table S1.

**Figure S1**

**
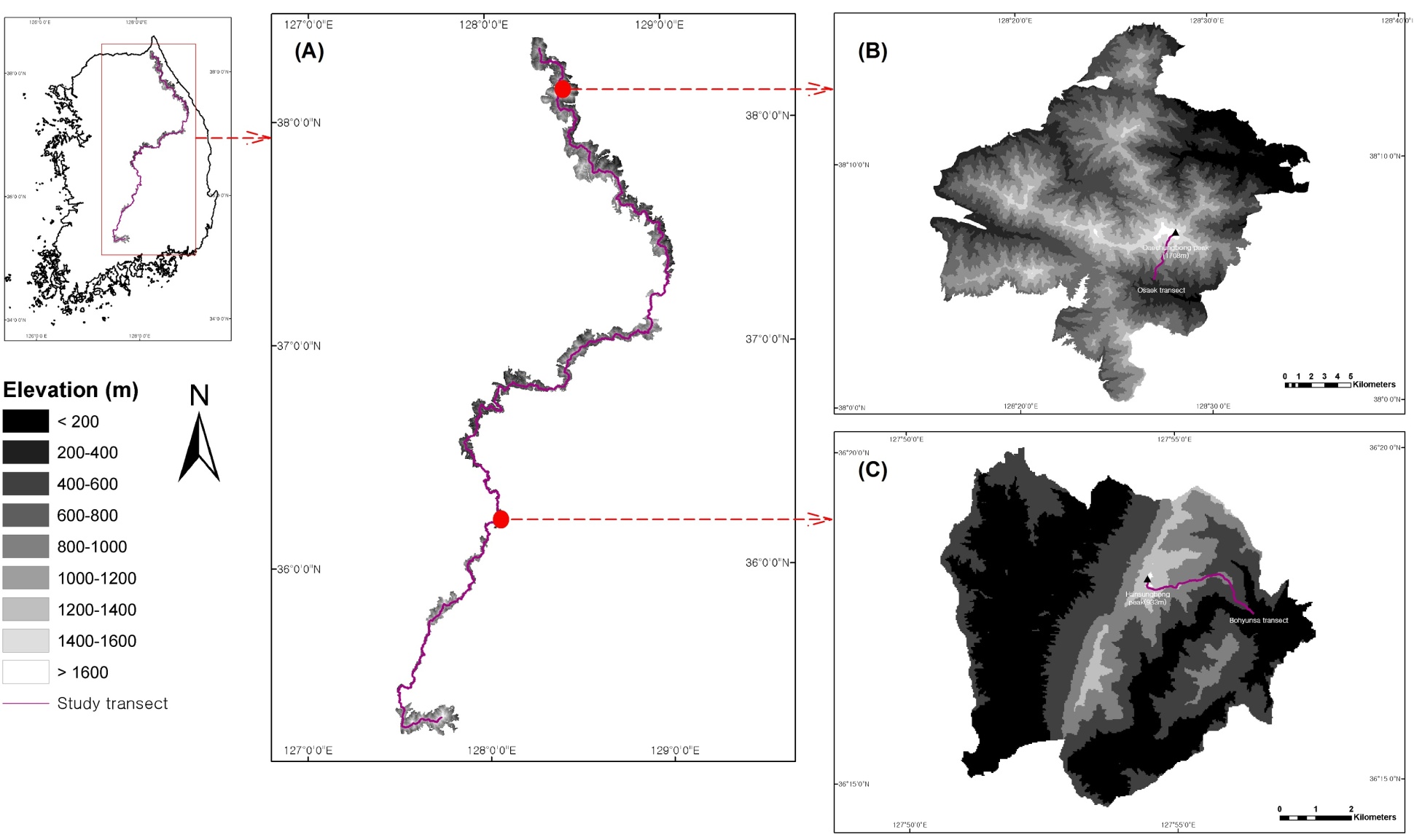
**

**Figure S2**

**
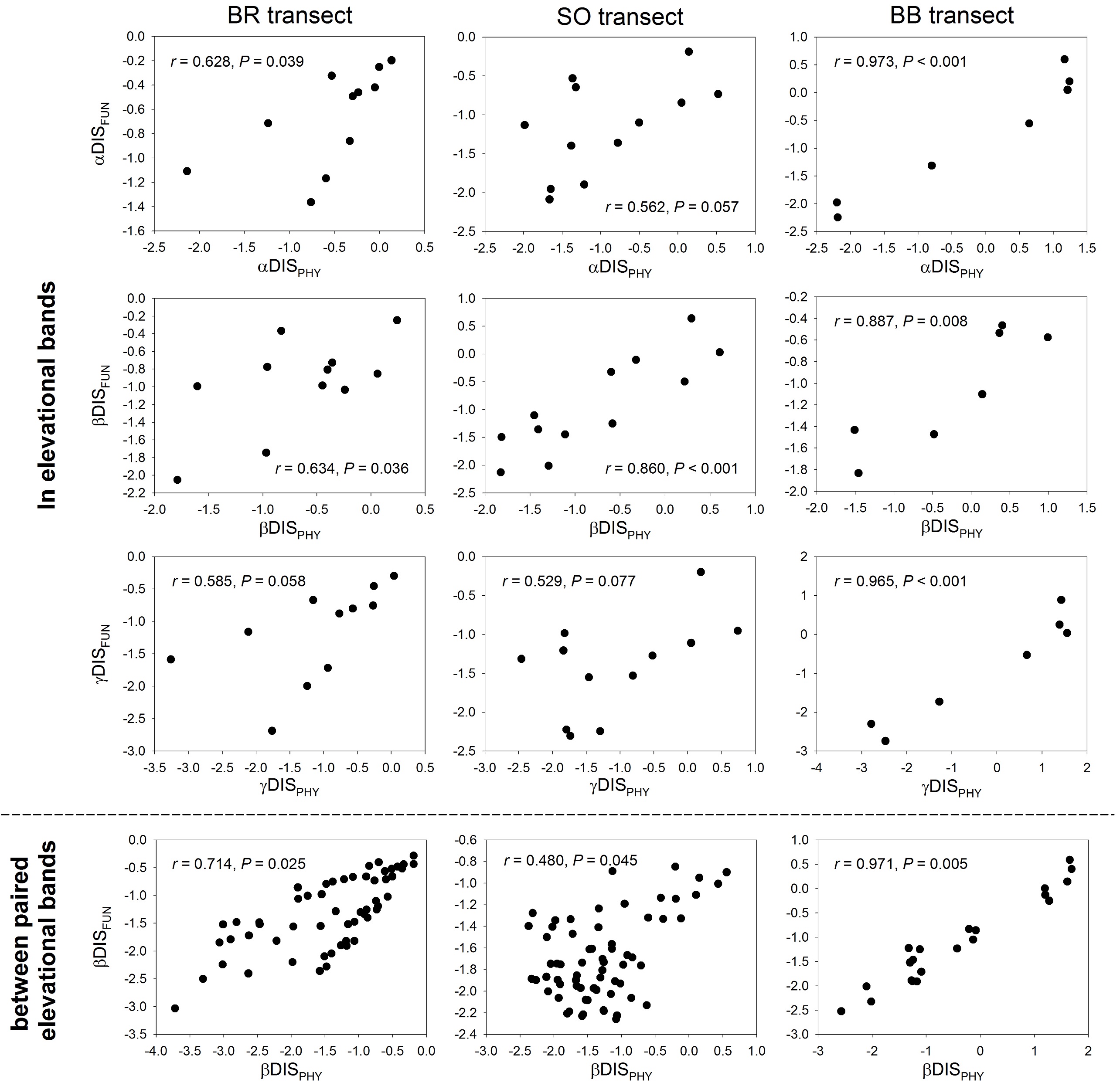
**

**Figure S3**

**
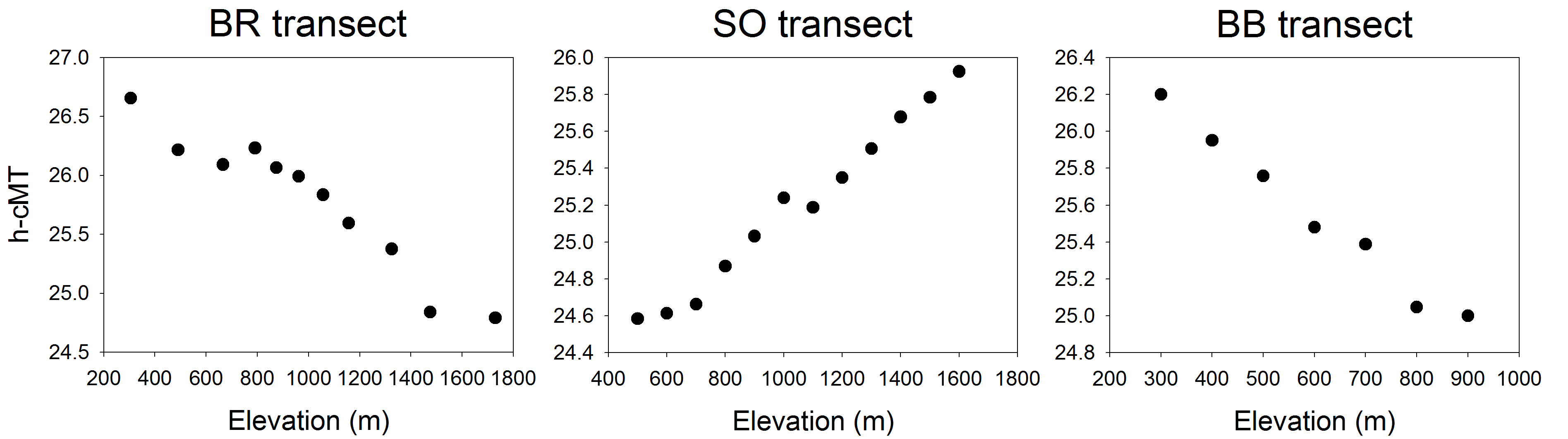
**

**Figure S4**

**(A) Phylogenetic tree_TOTAL_ (B) Functional trait dendrogram_TOTAL_**

**(C) Phylogenetic tree_BR_ (D) Functional trait dendrogram_BR_**

**(E) Phylogenetic tree_SO_ (F) Functional trait dendrogram_SO_**

**(G) Phylogenetic tree_BB_ (H) Functional trait dendrogram_BB_**

**Figure S5**


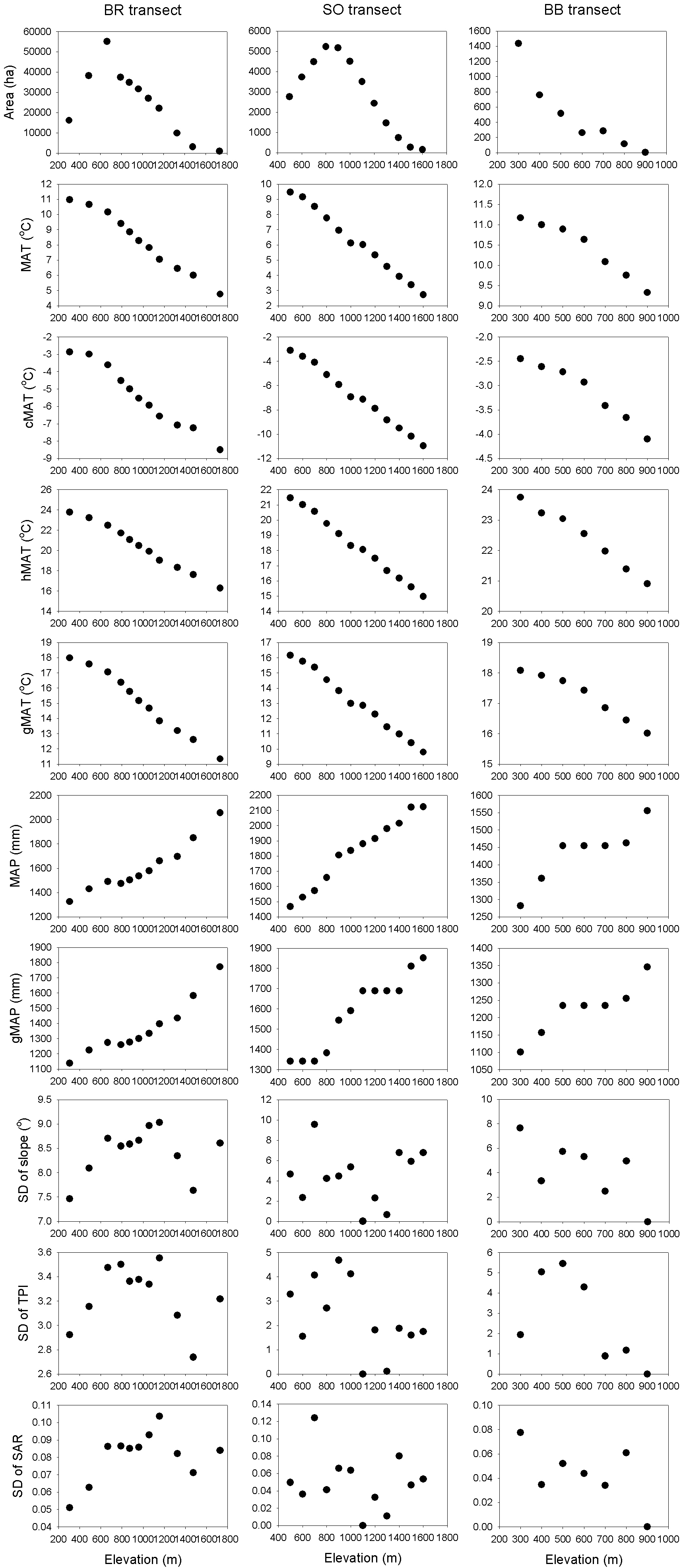


**List of Supplementary Tables**

**Table S1.** Number of elevational bands and elevational ranges defined along the three elevational transects in this study. The number in bracket indicates the number of plots established in each elevational band.

**Table S2.** Characteristics of the three elevational transects (with abbreviations) used in this study.

**Table S3.** Relationships between elevation and the three components (α, β and γ) of phylogenetic and functional diversity with linear and quadratic models along the three study transects in South Korea. DIS_PHY_ and DIS_FUN_ indicate phylogenetic and functional dispersion, respectively. The abbreviations for the study transects are defined in Table S1. * *P* < 0.05; ** *P* < 0.01; *** *P* < 0.001.

**Table S4.** Coefficients of determination (*R^2^*) and significance levels from simple conditional autoregressive models of the environmental variables and three components (α, β and γ) of phylogenetic and functional diversity derived in elevational bands along the three study transects. DIS_PHY_ and DIS_FUN_ indicate phylogenetic and functional dispersion, respectively. * *P* < 0.05; ** *P* < 0.01; *** *P* < 0.001

**Table S5.** Results of multiple conditional autoregressive (CAR) models of environmental variables or distance matrices and the three components (α, β and γ) of phylogenetic and functional diversity derived (a) in elevational bands and (b) between paired elevational bands along the three study transects. DIS_PHY_ and DIS_FUN_ indicate phylogenetic and functional dispersion, respectively. Multiple CAR models were performed only for the best model obtained based on forward stepwise multiple regression (FSMR) because the main purpose of using CAR models in this study was to assess the influence of spatial autocorrelation, such as inflation of type I error and invalid parameter estimation, on the results of the FSMR models. Variables with significant standardized regression slopes are identified from multiple CAR models. * *P* < 0.05; ** *P* < 0.01; *** *P* < 0.001

**Table S6.** Functional trait data of woody plant species used in this study and the frequencies of species found in each elevational band along the three study transects.

* Note: Table S6 was prepared from a different file (excel file). The table could not be included in these supplementary materials due to the large file size.

**Table S7.** Results of a principal component analysis of five functional traits.

**Table S8.** Results of two separate principal component analyses of climatic variables and the standard deviations (SDs) of topographic variables. MAP and gMAP of climatic variables were log-transformed before the PCA to achieve normality.

**Table S1.** Number of elevational bands and elevational ranges defined along the three elevational transects in this study. The number in bracket indicates the number of plots established in each elevational band.

| **Elevational band** | **Elevational range (m)** | | |
| --- | --- | --- | --- |
|  | **BR** | **SO** | **BB** |
| Band 1 | 210–400 (100) | 500–599 (5) | 300–399 (5) |
| Band 2 | 401–580 (100) | 600–699 (5) | 400–499 (5) |
| Band 3 | 581–750 (100) | 700–799 (5) | 500–599 (5) |
| Band 4 | 751–832 (100) | 800–899 (5) | 600–699 (5) |
| Band 5 | 833–916 (100) | 900–999 (5) | 700–799 (5) |
| Band 6 | 917–1005 (100) | 1000–1099 (5) | 800–899 (5) |
| Band 7 | 1006–1108 (100) | 1100–1199 (5) | > 900 (5) |
| Band 8 | 1109–1250 (100) | 1200–1299 (5) | - |
| Band 9 | 1251–1400 (100) | 1300–1399 (5) | - |
| Band 10 | 1401–1550 (100) | 1400–1499 (5) | - |
| Band 11 | > 1551 (100) | 1500–1599 (5) | - |
| Band 12 | - | > 1600 (5) | - |

Abbreviations: BR – Baekdudaegan ridge; SO – Osaek transect in Mt. Seorak; BB – Bohyunsa transect in Mt. Baekhwa.

**Table S2.** Characteristics of the three elevational transects (with abbreviations) used in this study.

| **Mountain** | **Study transect** | **Transect length (km)** | **Elevation extent (m)** | **Sampled domain (m)** | **Field sampling period** | **No. of elevational bands** | **No. of plots** | **No. of species** |
| --- | --- | --- | --- | --- | --- | --- | --- | --- |
| Baekdudaegan Mountains | Baekdudaegan ridge (BR) | 689.8 | 200–1909 | 210–1872 | May 2005–August 2009 | 11 | 1100 | 248 |
| Mt. Seorak | Osaek (SO) | 5.3 | 457–1708 | 507–1676 | May–July 2011 | 12 | 60 | 72 |
| Mt. Baekhwa | Bohyunsa (BB) | 4.1 | 255–933 | 301–925 | June–August 2011 | 7 | 35 | 58 |

**Table S3.** Relationships between elevation and the three components (α, β and γ) of phylogenetic and functional diversity with linear and quadratic models along the three study transects in South Korea. DIS_PHY_ and DIS_FUN_ indicate phylogenetic and functional dispersion, respectively. The abbreviations for the study transects are defined in Table S1. * *P* < 0.05; ** *P* < 0.01; *** *P* < 0.001.

| **Transect** | **Diversity index** | **First-order OLS** | |  | **Second-order OLS** | |
| --- | --- | --- | --- | --- | --- | --- |
|  |  | **AIC** | **R^2^** |  | **AIC** | **R^2^** |
| BR | αDIS_PHY_ | 27.130 | 0.243 |  | 8.133 | 0.916^***^ |
|  | βDIS_PHY_ | 29.659 | 0.010 |  | 13.102 | 0.863^***^ |
|  | γDIS_PHY_ | 37.029 | 0.148 |  | 17.491 | 0.910^***^ |
|  | αDIS_FUN_ | 18.149 | 0.119 |  | 1.384 | 0.920^**^ |
|  | βDIS_FUN_ | 21.998 | 0.279 |  | 15.978 | 0.741^**^ |
|  | γDIS_FUN_ | 25.592 | 0.250 |  | 8.596 | 0.931^***^ |
|  |  |  |  |  |  |  |
| SO | αNRI_PHY_ | 28.008 | 0.525^**^ |  | 32.593 | 0.530^*^ |
|  | βDIS_PHY_ | 31.943 | 0.384^*^ |  | 36.513 | 0.392 |
|  | γDIS_PHY_ | 32.455 | 0.540^**^ |  | 36.845 | 0.552^*^ |
|  | αNRI_FUN_ | 29.530 | 0.032 |  | 33.679 | 0.077 |
|  | βDIS_FUN_ | 35.402 | 0.220 |  | 38.360 | 0.326 |
|  | γDIS_FUN_ | 29.793 | 0.057 |  | 33.872 | 0.105 |
|  |  |  |  |  |  |  |
| BB | αDIS_PHY_ | 25.516 | 0.858^**^ |  | 35.163 | 0.924^**^ |
|  | βDIS_PHY_ | 14.410 | 0.922^***^ |  | 27.023 | 0.936^**^ |
|  | γDIS_PHY_ | 26.738 | 0.885^**^ |  | 36.510 | 0.937^**^ |
|  | αDIS_FUN_ | 16.981 | 0.916^***^ |  | 30.746 | 0.919^**^ |
|  | βDIS_FUN_ | 8.822 | 0.890^***^ |  | 21.625 | 0.907^**^ |
|  | γDIS_FUN_ | 21.098 | 0.902^***^ |  | 35.077 | 0.903^**^ |

**Table S4.** Coefficients of determination (*R^2^*) and significance levels from simple conditional autoregressive models of the environmental variables and three components (α, β and γ) of phylogenetic and functional diversity derived in elevational bands along the three study transects. DIS_PHY_ and DIS_FUN_ indicate phylogenetic and functional dispersion, respectively. * *P* < 0.05; ** *P* < 0.01; *** *P* < 0.001

| **Transect** | **Diversity index** | **RArea** | **PC1_clim_** | **PC1_hetero_** | **PC2_hetero_** |
| --- | --- | --- | --- | --- | --- |
| BR | αDIS_PHY_ | 0.782^***^ | 0.290 | 0.195 | < 0.001 |
|  | βDIS_PHY_ | 0.234 | < 0.001 | 0.560^**^ | 0.372^*^ |
|  | γDIS_PHY_ | 0.696^***^ | 0.188 | 0.290 | < 0.001 |
|  | αDIS_FUN_ | 0.041 | 0.074 | 0.529^*^ | 0.566^**^ |
|  | βDIS_FUN_ | < 0.001 | 0.207 | 0.488^*^ | 0.582^**^ |
|  | γDIS_FUN_ | < 0.001 | 0.189 | 0.567^**^ | 0.694^***^ |
| SO | αDIS_PHY_ | 0.393^*^ | 0.549^**^ | 0.172 | 0.207 |
|  | βDIS_PHY_ | 0.205 | 0.404^*^ | 0.256 | 0.207 |
|  | γDIS_PHY_ | 0.438^*^ | 0.568^**^ | 0.105 | 0.171 |
|  | αDIS_FUN_ | < 0.001 | 0.028 | 0.497^**^ | 0.171 |
|  | βDIS_FUN_ | 0.025 | 0.240 | 0.671^***^ | 0.094 |
|  | γDIS_FUN_ | < 0.001 | 0.051 | 0.489^*^ | 0.192 |
| BB | αDIS_PHY_ | 0.466 | 0.867^**^ | 0.427 | 0.133 |
|  | βDIS_PHY_ | 0.676^*^ | 0.903^***^ | 0.511 | 0.106 |
|  | γDIS_PHY_ | 0.492 | 0.888^**^ | 0.470 | 0.136 |
|  | αDIS_FUN_ | 0.629^*^ | 0.828^**^ | 0.593^*^ | 0.217 |
|  | βDIS_FUN_ | 0.488 | 0.818^**^ | 0.646^*^ | 0.150 |
|  | γDIS_FUN_ | 0.653^*^ | 0.815^**^ | 0.607^*^ | 0.232 |

Abbreviations: RArea – regional area with log-transformation; PC1_clim_ – PC1 from climatic variables; PC1_hetero_ – PC1 from standard deviations of topographic variables; PC2_hetero_ – PC2 from standard deviations of topographic variables. The abbreviations for the study transects are defined in Table S1.

**Table S5.** Results of multiple conditional autoregressive (CAR) models of environmental variables or distance matrices and the three components (α, β and γ) of phylogenetic and functional diversity derived (a) in elevational bands and (b) between paired elevational bands along the three study transects. DIS_PHY_ and DIS_FUN_ indicate phylogenetic and functional dispersion, respectively. Multiple CAR models were performed only for the best model obtained based on forward stepwise multiple regression (FSMR) because the main purpose of using CAR models in this study was to assess the influence of spatial autocorrelation, such as inflation of type I error and invalid parameter estimation, on the results of the FSMR models. Variables with significant standardized regression slopes are identified from multiple CAR models. * *P* < 0.05; ** *P* < 0.01; *** *P* < 0.001

(a) in elevational bands

| **Transect** | **Diversity index** | **Independent variables** | | | | **Model paramters** | |
| --- | --- | --- | --- | --- | --- | --- | --- |
|  |  | **RArea** | **PC1_clim_** | **PC1_hetero_** | **PC2_hetero_** | **F** | **R^2^** |
| BR | αDIS_PHY_ | 0.959^***^ | - | - | - | 32.201 | 0.782^***^ |
|  | βDIS_PHY_ | - | - | 0.667^*^ | - | 11.432 | 0.560^**^ |
|  | γDIS_PHY_ | 0.930^***^ | - | - | - | 20.571 | 0.696^***^ |
|  | αDIS_FUN_ | - | - | - | –0.793^**^ | 11.721 | 0.566^**^ |
|  | βDIS_FUN_ | - | - | - | –0.792^*^ | 12.548 | 0.582^**^ |
|  | γDIS_FUN_ | - | - | - | –0.836^**^ | 20.432 | 0.694^***^ |
| SO | αDIS_PHY_ | - | 0.755^*^ | - | - | 12.157 | 0.549^**^ |
|  | βDIS_PHY_ | - | 0.645^*^ | - | - | 6.770 | 0.404^*^ |
|  | γDIS_PHY_ | - | 0.772^*^ | - | - | 13.122 | 0.568^**^ |
|  | αDIS_FUN_ | - | - | 0.622^*^ | - | 9.878 | 0.497^**^ |
|  | βDIS_FUN_ | - | - | 0.739^***^ | - | 20.351 | 0.671^***^ |
|  | γDIS_FUN_ | - | - | 0.608^*^ | - | 9.579 | 0.489^*^ |
| BB | αDIS_PHY_ | - | –0.900^**^ | - | - | 32.477 | 0.867^**^ |
|  | βDIS_PHY_ | - | –0.915^**^ | - | - | 46.424 | 0.903^***^ |
|  | γDIS_PHY_ | - | –0.912^**^ | - | - | 39.455 | 0.888^**^ |
|  | αDIS_FUN_ | - | –0.853^**^ | - | –0.374^*^ | 47.134 | 0.959^**^ |
|  | βDIS_FUN_ | - | - | –0.847^*^ | - | 22.462 | 0.818^**^ |
|  | γDIS_FUN_ | - | –0.851^**^ | –0.386^*^ | - | 45.776 | 0.958^**^ |

(b) between paired elevational bands

| **Transect** | **Diversity index** | **Independent variables** | | | | **Model parameters** | |
| --- | --- | --- | --- | --- | --- | --- | --- |
|  |  | ***Dist_ele_*** | ***Dist_rarea_*** | ***Dist_clim_*** | ***Dist_habit_*** | **F** | **R^2^** |
| BR | βDIS_PHY_ | –0.374^***^ | –0.597^***^ | - | - | 80.108 | 0.755^***^ |
|  | βDIS_FUN_ | –2.446^***^ | - | 1.708^***^ | - | 48.899 | 0.653^***^ |
| SO | βDIS_PHY_ | - | –0.399^***^ | - | - | 20.279 | 0.241^***^ |
|  | βDIS_FUN_ | - | - | –0.250^*^ | –0.214^*^ | 4.440 | 0.124^*^ |
| BB | βDIS_PHY_ | - | 0.404^*^ | –0.736^***^ | - | 10.797 | 0.545^***^ |
|  | βDIS_FUN_ | - | 0.461^*^ | –0.623^***^ | - | 7.637 | 0.459^**^ |

Abbreviations: *Dist_ele_* – elevation difference; *Dist_rarea_* – regional area distance; *Dist_clim_* – climate distance; *Dist_habit_* – habitat heterogeneity distance. The abbreviations for the study transects, environmental variables and diversity indices are defined in Table S2 and S3.

**Table S7. Results of a principal component analysis of five functional traits. Values of all traits were log transformed to improve normality and to standardize before analysis.**

|  | **PC1** | **PC2** | **PC3** | **PC4** | **PC5** |
| --- | --- | --- | --- | --- | --- |
| Functional traits data |  |  |  |  |  |
| Explained variance (%) | 39.674 | 24.572 | 18.027 | 12.569 | 5.158 |
| Loadings |  |  |  |  |  |
| Maximum height (m) | 0.405 | -0.532 | 0.255 | 0.621 | 0.320 |
| Leaf length (cm) | 0.621 | 0.231 | –0.121 | 0.209 | –0.709 |
| Leaf width (cm) | 0.499 | 0.507 | –0.303 | –0.090 | 0.627 |
| Seed weight (mg) | 0.442 | –0.310 | 0.423 | –0.727 | 0.000 |
| Flowering onset (month) | –0.079 | 0.557 | 0.806 | 0.183 | 0.028 |

**Table S8.** Results of two separate principal component analyses of climatic variables and the standard deviations (SDs) of topographic variables. MAP and gMAP of climatic variables were log-transformed before the PCA to achieve normality.

|  | **PC1** | **PC2** | **PC3** | **PC4** | **PC5** | **PC6** |
| --- | --- | --- | --- | --- | --- | --- |
| Climatic variables |  |  |  |  |  |  |
| Explained variance (%) | 97.555 | 1.900 | 0.376 | 0.115 | 0.049 | 0.004 |
| Loadings |  |  |  |  |  |  |
| MAT | 0.411 | 0.250 | 0.069 | 0.277 | –0.601 | 0.571 |
| cMAT | 0.406 | 0.459 | –0.714 | 0.024 | 0.330 | –0.071 |
| hMAT | 0.411 | 0.184 | 0.575 | –0.271 | 0.563 | 0.277 |
| gMAT | 0.412 | 0.212 | 0.328 | 0.127 | –0.265 | –0.769 |
| MAP | –0.408 | 0.448 | 0.207 | 0.717 | 0.277 | 0.016 |
| gMAP | –0.402 | 0.669 | 0.063 | –0.566 | –0.258 | –0.008 |
|  |  |  |  |  |  |  |
| Topographic heterogeneity variables |  |  |  |  |  |  |
| Explained variance (%) | 79.362 | 18.247 | 2.391 | - | - | - |
| Loadings |  |  |  |  |  |  |
| SD of slope | 0.614 | –0.349 | –0.708 | - | - | - |
| SD of topographic position index | 0.497 | 0.868 | 0.003 | - | - | - |
| SD of surface area ratio | 0.613 | –0.354 | 0.706 | - | - | - |

Abbreviations: MAT – mean annual temperature; cMAT – mean temperature in the coldest month (January); hMAT – mean temperature in the hottest month (August); gMAT – mean temperature of the growing season (May-August); MAP – mean annual precipitation; gMAP – mean precipitation of the growing season.
